# Pulmonary Cystic Disease Associated With COVID 19 Pneumonia: An Emerging Atypical Manifestation

**DOI:** 10.7759/cureus.19352

**Published:** 2021-11-08

**Authors:** Bernardo J Muñoz-Palacio, Daniel Syro, Miguel A Pinzón, Beatriz Ramirez, Juan F Betancur

**Affiliations:** 1 Pulmonology, Clínica Medellín/Grupo QuirónSalud, Medellín, COL; 2 Anesthesiology and Reanimation, CES University, Medellín, COL; 3 Infectious Disease, Clínica Medellín/Grupo QuirónSalud, Medellín, COL; 4 Epidemiology, Clínica Medellín/Grupo QuirónSalud, Medellin, COL; 5 Internal Medicine, Clínica Medellín/Grupo QuirónSalud, Medellín, COL; 6 Internal Medicine, Sura, Medellín, COL

**Keywords:** covid-19, cystic lesions of the lung, atypical coronavirus, invasive mechanical ventilation, severe acute respiratory syndrome coronavirus-2 (sars-cov-2)

## Abstract

Chest tomography has played an essential role during the coronavirus disease 2019 (COVID-19) pandemic since it has allowed to suspect and diagnose the disease early and to assess the severity of lung involvement, predict the disease's course, and detect the complications associated with it. Certain chest CT findings have been reported in more than 70% of reverse transcription polymerase chain reaction (RT-PCR) test-proven COVID-19 cases, including ground-glass opacities, vascular enlargement, bilateral abnormalities, lower lobe involvement, and posterior predilection. In COVID-19-endemic regions, observing these chest CT findings should raise the suspicion of a possible COVID-19 diagnosis. Rare reported CT findings in RT-PCR test-proven COVID-19 cases include pleural effusion, lymphadenopathy, tree-in-bud sign, central lesion distribution, pericardial effusion, and cavitating lung lesions. The observation of one or more of these findings suggests an alternative diagnosis, although COVID-19 cannot be excluded from the differential diagnosis. Here, we report an interesting case of a patient with no relevant history presenting a COVID-19 infection which, as a complication, presented cystic lesions; we discuss its etiology briefly.

## Introduction

Coronavirus disease 2019 (COVID-19) is caused by the severe acute respiratory syndrome coronavirus 2 (SARS-CoV2) virus, an emerging pathogen initially identified in Wuhan, China, December 2019 [1]. Until July 5, 2021, 183.949.434 people are infected globally, with 3.979.944 deaths [2]. Chest tomography has played a fundamental role during the COVID-19 pandemic. With a sensitivity of 89%-97%, it has allowed to suspect and diagnose the disease before the test is positive, it aids to predict the disease's course, and detect the complications associated with it [3,4].

In more than 70% of RT-PCR test-proven COVID-19 cases, several chest CT typical findings are reported, including ground-glass opacities, vascular enlargement, bilateral abnormalities, lower lobe involvement, and posterior predilection [3,5]. In COVID-19-endemic regions, observing these chest CT findings should raise the suspicion of possible COVID-19 diagnosis [3,5].

With the passing of the pandemic, the spectrum of less frequent radiological manifestations (incidence < 10%) has been described and increased in RT-PCR COVID-19 confirmed cases. These include pleural effusion, lymphadenopathy, tree-in-bud sign, central lesion distribution, pericardial effusion, and cavitating lung lesions [3,5]. The isolated observation of one or more of these findings suggest another diagnosis than COVID-19, although it cannot be ruled out [3,5].

The clinician must recognize the early and typical manifestations of the disease and keep in mind the atypical manifestations and complications associated with it, in order to establish their associated factors, prognosis, and probability of long-term recovery.

## Case presentation

**Figure 1:**
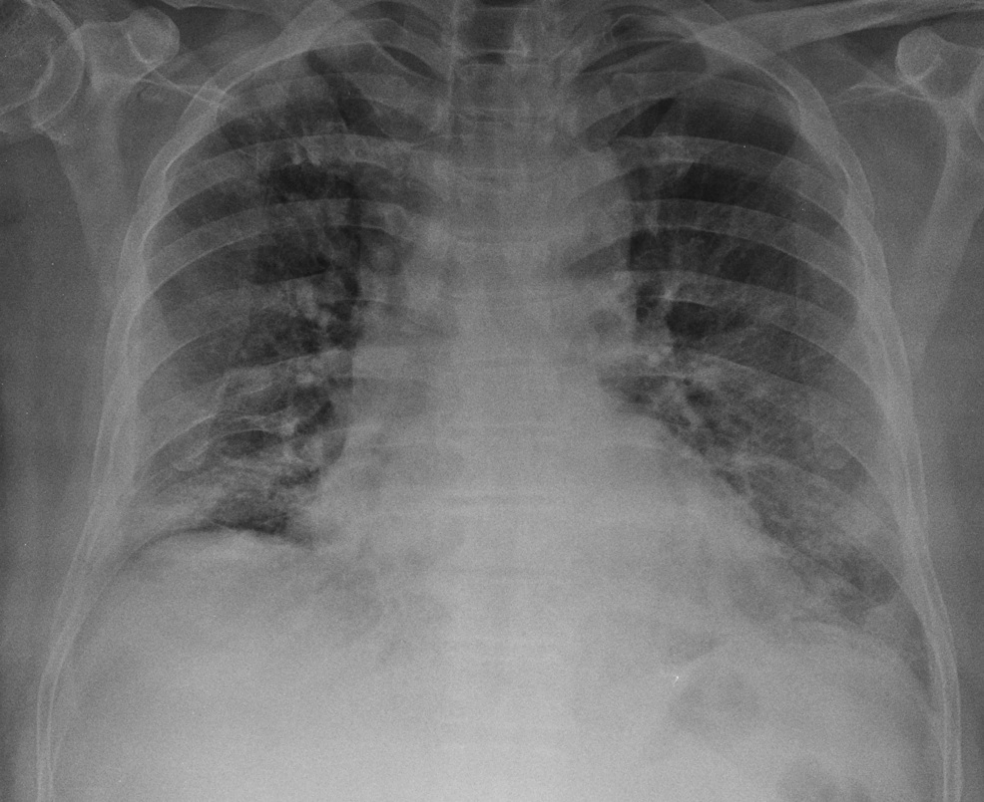
Chest X-ray of the patient on admission. Chest X-ray: peripheral patchy air space opacification in lower zones with diffuse ground opacities bilaterally.

**Figure 2:**
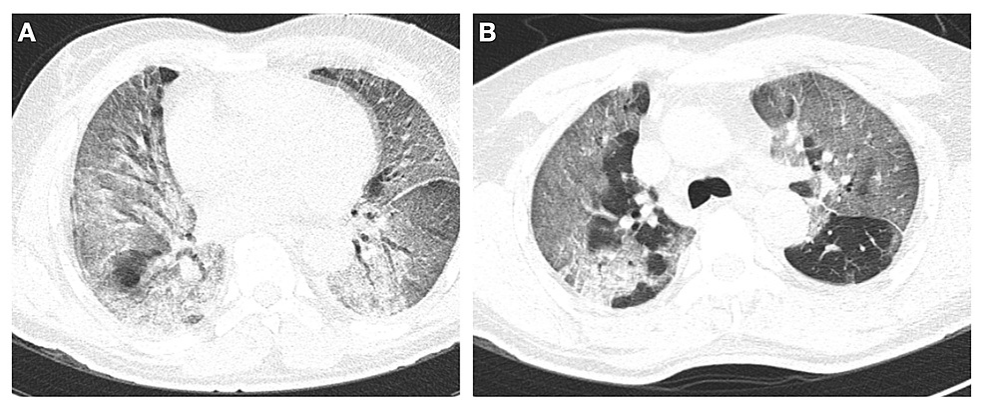
Chest CT of the patient on admission. Chest CT coronal (A) air entrapment areas, with crazy paving and diffuse bilateral ground-glass opacities, consolidation in the posterior segment of the right upper lobe. Chest CT coronal (B) large bilateral areas of ground-glass opacities and consolidation with air bronchogram in both lungs. Crazy paving in the lingula and medium lobe.

**Figure 3:**
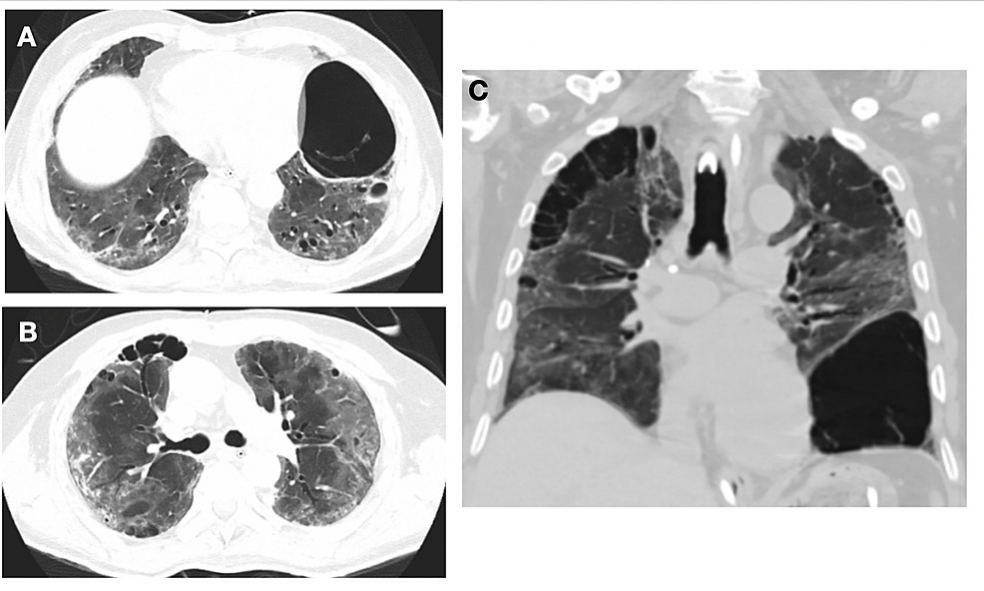
Pulmonary cystic changes associated with COVID-19. Chest CT coronal (A): Fibrotic appearance changes with increased density of the lung parenchyma and thickening of the central and peripheral interstitium, bronchial thickening, ground-glass opacities, and cystic lesions. Chest CT coronal (B): Cystic changes of paraseptal emphysema-like in the right upper lobe, ground-glass opacities, and bronchial thickening. Chest CT sagittal (C). Large left basal cyst measuring 70 x 84 mm. Cystic changes in right upper lobe bronchial thickening and ground-glass opacities.

A 56-year-old male with no relevant history presented at the emergency department with five days of malaise, fever, cough, chest pain, and shortness of breath. SARS-CoV-2 infection was suspected. Admission Blood tests showed severe hypoxemia in arterial blood gases, leukocytosis, and elevated acute phase reactants; the SARS-CoV-2 RT- PCR was positive. The admission chest X-ray (Figure 1) and CT (Figure 2) revealed generalized, bilateral, and patchy ground-glass opacities. The patient's clinical condition worsened and developed an acute respiratory syndrome (ARDS) with respiratory failure type I and he was transferred to the ICU. The patient required the support of mechanical ventilation for the next ten weeks. A control chest CT was performed two months later, disclosing bilateral lung cystic lesions and bronchiolitis obliterans organizing pneumonia (Figure 3). There were no bacterial superinfections. After three months in the ICU, the patient was transferred to the general wards and discharged a week after with oxygen by nasal cannula and oral steroids with progressive tapering until discontinuation. In his 3-month follow-up and to this day, the patient is in acceptable general conditions, the supplemental oxygen was weaned, and he has not presented pneumothorax or emergency visits.

## Discussion

Coronavirus disease 2019 is caused by the SARS-CoV-2. This disease usually presents multiorgan clinical and radiological involvement, with a particular predilection for the lungs. Its spectrum ranges from asymptomatic to ventilatory failure. Chronologically, on chest CT in the first five days, ground-glass opacities, mixed infiltrate of ground-glass, and consolidation, with subpleural and peripheral in location are observed, with a peak of these findings around day 10 [6]. Other frequent chest CT findings in COVID-19 are vascular enlargement, crazy paving, interlobular septal thickening, and air bronchogram [5,7,8]. Recently, a collection of emerging radiological findings has been categorized as atypical: subsegmental pulmonary vessels enlargement (> 3 mm), halo sign, reversed halo sign, cavitation, pleural or pericardial effusion, and pulmonary cysts [5,7,8]. Some of these findings are temporally related to the disease duration, manifesting later in the course of the infection.

According to clinical evolution, the tomographic manifestations can take different directions in the acute infection. In some patients, ground-glass opacities and consolidations can progress to mild focal reticular abnormalities by week two, and consolidation increase by week three [6]. In patients with milder infections, it is from third week that the tomographic findings begin to resolve, completing towards the first or second month [6]. In patients with disease progression, post-acute fibrosis can develop, with persistent symptoms or complications beyond the fourth week of the onset of symptoms, configuring what is now called the post-acute sequelae (PASC) COVID-19 [6]. Radiologically, these patients may present persistent abnormalities such as interlobular septal thickening, focal, and multifocal ground-glass opacities. Other reported findings are subpleural parenchymal bands and signs of fibrosis: thin fibrous bands with or without parenchymal distortion, bronchiectasis, bronchioloectasis, areas of decreased attenuation due to small airway disease, emphysematous or cystic areas [6].

The etiology of the post-acute covid lung is attributed to acute respiratory distress syndrome (ARDS), mechanical ventilation (Barotrauma), or direct virus injury [6].

Predictors of the PASC COVID-19 lung are at the clinical level: advanced age, male, comorbidities, severe COVID that requires hospitalization, oxygen, transfer to ICU, and mechanical ventilation. Laboratory parameters include elevated acute phase reactants or prognostic factors: Leukocytosis, C-reactive protein, LDH, IL-6, and elevated D-dimer. Radiologically: the extent of the initial tomographic abnormality [6].

Regarding pulmonary cysts, their diagnosis is challenging; it is essential to distinguish cysts from cavities because they have different etiologies and associated clinical disorders. Cyst occurs in greater profusion in the subpleural areas, typically representing emphysema, bullae, or honeycombing [8,9]. The descriptions of cystic disease related to COVID 19 are unusual, with a prevalence ranging between 9% and 25% [10]. They are usually well-defined, thin-walled (2-4 mm), and variable in size, but usually less than 2.5 cm; larger cysts have been described [10,11]. They are usually distributed peripherally and sometimes have a random or perihilar distribution; it has been found in different series a predominance in the anterior part of the lung and the lower lobe, followed by the upper and middle lobe [10,11]. The presence of cysts increases the specificity of the diagnosis since these findings are not reported in other viral pneumonia, as long as the patient has not had pre-existing emphysema or interstitial disease [11]. It must also be differentiated from the patient with pre-existing cystic lesions with co-existence of COVID 19 as the patient with previous cystic bronchiectasis, emphysematous bullae, and pneumatoceles [11].

To date, its etiology is merely speculative, which is presumed to be secondary to parenchymal damage, pulmonary fibrosis, or low compliance, in some cases associated with mechanical ventilation but can also arise spontaneously in intermediate and late stages of ARDS; others assert that it is associated with the process of resorption of consolidation [12-19]. However, it is interesting to highlight that the infection by COVID-19 and other coronaviruses produces persistent airflow obstruction since it is common to find new emphysema, cysts, or attenuation mosaic in these infections, which is not the case with other viral infections [20].

Physiopathologically it is postulated that this type of lesion is formed in response to mucus plugs, or fibromyxoid exudates, with a valve effect in the bronchus. The persistent cough might trigger a sudden increase in the intra-alveolar pressure, inducing subpleural bullae or pneumothorax formation if the alveoli rupture [13]. This complication should be suspected if the patient has worsening dyspnea, absent breath sounds, and a deteriorating clinical condition [17,18]. Regarding the treatment or the prevention of its appearance, it is unknown whether, unlike organized pneumonia and post-covid persistent interstitial disease, if steroids prevent or accelerate the resolution of cystic lung lesions [6]. It is recommended that if the patient presents any of the risk factors associated with post-acute covid or cystic lesions, a CT scan with expiratory phase is performed [6].

## Conclusions

With the course of the pandemic, we increasingly see atypical pulmonary radiological manifestations of the disease that may manifest in the late course of the disease, which usually occurs in patients who had severe pulmonary involvement with elevated inflammatory markers, ARDS requiring ICU and mechanical ventilation with difficult weaning. We could categorize cystic lung lesions as radiological manifestations of the post-acute COVID spectrum whose etiology is presumed to be secondary to direct injury by the virus, secondary to COVID-induced ARDS, or sequelae of mechanical ventilation or its prolonged duration. Unlike the other post-acute complications, the rupture of a cystic lesion with subsequent pneumothorax and pneumomediastinum, a life-threatening complication occurring in almost 15% of the patients and should be borne in mind in a patient with risk factors. Only the passing of time and future research is needed to determine the true prevalence and persistence of post-COVID lung disease as its management and prevention.
